# Self-management and role of nurses of diabetic patients: a critical narrative literature review during COVID-19 pandemic

**DOI:** 10.3389/fmed.2025.1626447

**Published:** 2025-08-28

**Authors:** Zhixiong Zhuang, Yan Bo

**Affiliations:** ^1^Intensive Care Unit, Ningbo Sixth Hospital, Ningbo, Zhejiang, China; ^2^The Department of Medicine, Northwest Minzu University, Lanzhou, Gansu, China

**Keywords:** COVID-19, diabetes, health management, nurses, nursing education

## Abstract

**Background:**

During COVID-19 and the postepidemic phase, the concept of a frontline emergency response team was gradually expanded [based on a previous clinical trial, https://clinicaltrials.gov/, identifier (ChiCTR2500103976)]. The professional scope of nurses gradually expanded towards the digital. We selected the digital interventions implemented by nurses during the COVID-19 pandemic and self-management by diabetes patients as the research context.

**Aims:**

To synthesise a framework for the module-intensity-outcome of digitalised care during COVID-19.

**Methods:**

We searched the PubMed database for literature related to the role of nurses and self-management of diabetes patients during the COVID-19 pandemic, and then used these literature as ‘seed literature’ for a snowball search. We synthesised all the evidence using a directional evidence synthesis method, a narrative description method, and a visual presentation method.

**Results:**

A total of 10 publications were retrieved from the PubMed database, involving a cumulative total of 5,834 adults. This indicates that using these 10 ‘seed literature’ for secondary literature retrieval enhances the persuasiveness of this method. Using these ‘seed literature’, an additional 28 literature were retrieved using the snowball method. Digital nursing has developed rapidly during the COVID-19 pandemic due to challenges in patient self-management. During the COVID-19 pandemic, patients sought digital nursing services from nurses due to disrupted healthcare services, surging mental health issues, and unhealthy lifestyles. In this model, nurses serve as a bridge between digital nursing services, the healthcare system, and patients. Digital nursing services not only bridge the gap in nursing services during healthcare crises but also expand the scope of nurses’ professional roles.

**Conclusion:**

Nurse-led digital care with a clear mix of “modules and intensity” may have directional benefits for diabetes self-management and metabolic outcomes.

## 1 Introduction

Diabetes has become a growing global health issue, with the number of people with diabetes increasing significantly over the past 35 years. According to statistics from the International Diabetes Federation (IDF), approximately 425 million adults worldwide had diabetes, and this number is expected to rise to 629 million by 2045. It is estimated that the prevalence of diabetes has risen more rapidly in low- and middle-income countries than in high-income countries over the past decade. Adults diagnosed with diabetes are 3.5 times more likely to be hospitalized than those without a history of diabetes, while those with prediabetes are 1.3 times more likely to be hospitalized than those without a history of diabetes (1).

In the face of the steadily increasing global number of diabetes patients, we need to find a new approach to address the challenges faced by diabetes patients. During the COVID-19 pandemic from 2019 to 2022, we observed a phenomenon: diabetes patients, unable to access hospital services in a timely manner, actively sought assistance from nurses in the COVID-19 frontline response teams. The reason behind this phenomenon is that contracting COVID-19 increases the likelihood of severe complications for diabetes patients (2). In other words, once diabetic patients contract COVID-19, they face a heavy medical burden and the threat of death. A report indicates that diabetic patients account for the second-highest number of COVID-19 infections (3). Another report states that diabetic patients have a twofold higher risk of severe illness compared to non-diabetic patients among COVID-19 patients (4). These statistics suggest that diabetic patients must learn to self-manage to avoid the heavy burden of contracting COVID-19. However, during the global spread of COVID-19, the public implemented public health measures such as social distancing and community lockdowns to interrupt transmission. These public health measures posed challenges for diabetes patients who rely on various social and environmental resources to manage their blood sugar levels, potentially altering their previous lifestyle habits (5).

As we delved deeper into our observations, we found that nurses strengthened their nursing practices while providing digital nursing services during the COVID-19 pandemic, which indirectly led to the development of unusual medical services such as digital nursing and telemedicine (2, 6). Nurses demonstrated greater patience in guiding diabetes patients in self-management. Therefore, we selected the role of nurses during the COVID-19 pandemic and diabetes patients’ self-management as the research context to explore the benefits of digital nursing task modules and intervention intensity on diabetes patients’ self-management.

## 2 Methods

### 2.1 Research purpose

This study was derived from a research concept proposed by Yan Bo in 2022: Health Needs Assessment of Emergency Frontline Rescuers. The study concept, design, and assessment details can be viewed in our research protocol (ChiCTR2500103976) published in 2025 (7).

While performing the research protocol during the COVID-19 pandemic in 2022, Yan Bo found that nurses who volunteered to be emergency frontline responders took on additional tasks when confronted with diabetic patients, thereby helping diabetic patients to self-manage. At the same time, patients with diabetes volunteered to avoid COVID-19 infection at home, a process equivalent to not being able to receive diabetes care in a hospital, which would promote patient self-management.

Based on this, Yan Bo extended the topic of this research protocol. The purpose of this study was to explore the self-management of patients with diabetes and the role of nurses during COVID-19. The added value of this study is to expand the boundaries of nurses’ management of patients with diabetes, a novel management concept that could be used in future unexpected public crises.

### 2.2 Research design

Our aim was to identify challenges, adaptations and effective practices in nursing under pandemic conditions. Such a broad topic is more appropriately designed as a narrative review. To ensure that the literature obtained through the narrative review process was open, transparent, and replicable, Yan Bo initially used the research framework of systematic reviews (PRISMA guidelines) (8) and combined it with a snowballing strategy to obtain literature. This mixed research methodology was gradually explored and developed by Yan Bo in the course of his past research (9).

### 2.3 Eligibility criteria

*Inclusion criteria*: (1) Studies involving adults (≥18 years) diagnosed with diabetes (ICD-11 criteria). (2) Literature addressing self-management practices or nursing roles during COVID-19. (3) Peer-reviewed articles published after 1 January 2020.

*Exclusion criteria*: (1) Studies focusing on non-diabetic populations. (2) Pre-pandemic studies or non-COVID-19 contexts.

### 2.4 Information sources

Our pre-set boundary was a narrative mini-review. To accomplish this goal, Yan Bo decided to adopt the previous strategy of searching only PubMed databases. Here, Yan Bo describes in detail the reasons and advantages of searching only PubMed.

The concept of using the PubMed database of literature to accomplish the planned tasks was first reported by Yan Bo in the 2022 international conference and then officially published in 2023 (10, 11). PubMed is an authoritative database of medical topics. This not only excludes low-quality studies, but also focuses research on medical topics.

Yan Bo in 2024 argues that literature-based research completed using the PubMed database should be written with a replicable methodology (12). This means that the thinking, literature, and the framework of ideas of a narrative review can be replicated by writing methods to replicate the study, avoiding subjectivity and non-verifiability.

Subsequently, Yan Bo (13) argued that narrative reviews might be more persuasive if they could go beyond text-based descriptions of viewpoints, and subsequently wrote a set of research protocols framed in a scoping review. Since most narrative reviews are based on research thinking, research perspectives, and research frameworks rather than data per se, overly arbitrary execution of data extraction, quality assessment, and publication bias assessment can undermine the rationale and core purpose of narrative review design. Based on this, Yan Bo has successfully applied narrative synthesis as an external validation tool for original research in several studies in his subsequent research practice (14–16). In all three cases, Yan Bo implemented a strategy of searching only PubMed databases, which allowed for more efficient completion of the research and delivery of up-to-date research information to the world. This strategy earned high praise from several reviewers. Subsequently, Yan Bo designed research protocols with the addition of an external validation session of the narrative review (7). This not only increased study credibility, but also avoided duplication after systematic review of similar studies. This ultimately results in a systematic opinion that is more persuasive and disseminated than the conventional single conclusion of the original study.

Interestingly, Yan Bo’s previous research in formulating systematic reviews and meta-analyses found that after removing duplicates and initial screening of the literature using multi-database searches such as PubMed, Embase, Web of Science, etc., the retained literature was almost exclusively from the PubMed database (17–19). Narrative reviews mainly express to propose new research hypotheses, present novel research ideas, and propose future research concepts, however, the purpose of systematic reviews is to superimpose the evidence and assess the heterogeneity of the evidence, and there is a fundamental difference between the two purposes. Based on these research practices, the source of information used in this study remains the PubMed database only.

### 2.5 Search

The time frame of the search is clearly stated (searches were run on 1 May 2022, 10 May 2025, and 5 August 2025).

#### 2.5.1 Search strategy

In order to achieve a replicable process for obtaining literature, this study followed a systematic review of search strategy development. These details can be viewed in previously published studies (17–19).

We distilled the core terms “diabetes,” “nurse,” “nursing,” “self-management” and “COVID-19” from the themes and topics of the study. We then entered these words into the PubMed database to MeSH terms and Entry terms. We found that “nurse” and “nursing” had similar meanings in the context of our study and were therefore merged. Based on this, we developed a search strategy for use in the PubMed database:

#1: ((“Nursing”[Mesh]) OR (Nursings[Title/Abstract])) OR (self-health management[Title/Abstract]))
#2: (((((((((((((((((((((((((((((((((((((“COVID-19”[Mesh]) OR (COVID-19[Title/Abstract])) OR (COVID 19[Title/Abstract])) OR (2019-nCoV Infection[Title/Abstract])) OR (2019 nCoV Infection[Title/Abstract])) OR (2019-nCoV Infections[Title/Abstract])) OR (Infection, 2019-nCoV[Title/Abstract])) OR (SARS-CoV-2 Infection[Title/Abstract])) OR (Infection, SARS-CoV-2[Title/Abstract])) OR (SARS CoV 2 Infection[Title/Abstract])) OR (SARS-CoV-2 Infections[Title/Abstract])) OR (2019 Novel Coronavirus Disease[Title/Abstract])) OR (2019 Novel Coronavirus Infection[Title/Abstract])) OR (COVID-19 Virus Infection[Title/Abstract])) OR (COVID 19 Virus Infection[Title/Abstract])) OR (COVID-19 Virus Infections[Title/Abstract])) OR (Infection, COVID-19 Virus[Title/Abstract])) OR (Virus Infection, COVID-19[Title/Abstract])) OR (COVID19[Title/Abstract])) OR (Coronavirus Disease 2019[Title/Abstract])) OR (Disease 2019, Coronavirus[Title/Abstract])) OR (Coronavirus Disease-19[Title/Abstract])) OR (Coronavirus Disease 19[Title/Abstract])) OR (Severe Acute Respiratory Syndrome Coronavirus 2 Infection[Title/Abstract])) OR (COVID-19 Virus Disease[Title/Abstract])) OR (COVID 19 Virus Disease[Title/Abstract])) OR (COVID-19 Virus Diseases[Title/Abstract])) OR (Disease, COVID-19 Virus[Title/Abstract])) OR (Virus Disease, COVID-19[Title/Abstract])) OR (SARS Coronavirus 2 Infection[Title/Abstract])) OR (2019-nCoV Disease[Title/Abstract])) OR (2019 nCoV Disease[Title/Abstract])) OR (2019-nCoV Diseases[Title/Abstract])) OR (Disease, 2019-nCoV[Title/Abstract])) OR (COVID-19 Pandemic[Title/Abstract])) OR (COVID 19 Pandemic[Title/Abstract])) OR (COVID-19 Pandemics[Title/Abstract])) OR (Pandemic, COVID-19[Title/Abstract])
#3: ((((((“Diabesity”[Mesh]) OR (Diabetes-Related Obesity[Title/Abstract])) OR (Diabetic Obesity[Title/Abstract])) OR (Obesity-Induced Diabetes[Title/Abstract])) OR (Obesity-Related Diabetes[Title/Abstract])) OR (“Diabetes Mellitus”[Mesh])) OR (diabetes[Title/Abstract])
#4: #1 AND #2 AND #3

Yan Bo ran the search on 1 May 2022 and found that only 6 literatures existed. on 10 May 2025 Yan Bo ran the search and found that only 9 literatures existed. Yan Bo then allowed the search for the last time on 5 August 2025 and found that only 10 literatures existed. As the purpose of the study is not to systematically review topics in a particular field, but to present a set of insights that might be applied to future public crises. This means that we may need more literature in order to gain perspectives, thinking, and a framework for the future.

All the literatures obtained from the initial search were served as “seed literatures” for the subsequent snowballing method.

#### 2.5.2 Snowballing strategy

Although some studies in the literature consider it important to retrieve all the literature that needs to be included in the study at the outset (17–19), we believe that the focus on a specific topic should be on step-by-step reasoning rather than being comprehensive and systematic. Based on this consideration, we will incorporate the retrieved literature related to self-management of diabetic patients during COVID-19 in the PubMed database according to the search strategy beforehand. More relevant literature is then progressively included using a snowballing strategy based on the need for stepwise reasoning. The snowballing strategy Yan Bo was previously successfully applied in investigating the public’s health needs for an AI platform during COVID-19 (20). Based on the stepwise inference process using the snowballing method to obtain literature, which Yan Bo has used in previous studies (10, 11). This strategy is more likely to find critical literature than random or comprehensive searches.

Since the snowballing method of obtaining literature was developed based on a previous study by Yan (20). Sufficient detail will be given here. We had to take the lead in developing strategies that would allow us to obtain, on the first attempt, precise literature related to the role of nurses and self-management for people with diabetes during COVID-19. These first-time acquisitions will serve as seed literature for the snowball method. In bibliographic method studies, the method of obtaining precise literature is generally an intentional search for specific keywords, but we chose the systematic review method of retrieving literature in order to obtain a sufficiently comprehensive seed literature. The retrieved literature must be included in the study in its entirety irrespective of its size, thus ensuring a sufficient number of seed literature. This is because the literature obtained by the systematic review method is dependent on the intentionally selected keywords for a comprehensive or systematic search.

Although some systematic reviews of the research reporting process have argued that only randomized controlled studies can be of interest to the researcher (17–19), for narrow topics even if a literature review presents the idea of the role of specific nurses and self-management in people with diabetes, that would be valuable. For this reason we did not qualify the type of literature or research methods. According to PRISMA guidelines (8), a systematic review is complete once a systematic search is performed and the evidence is superimposed. The systematic review at this point, due to limitations dependent on the search strategy, only indicates systematic within the current researcher’s perspective. This may result in the inclusion of studies that are underrepresented in the literature or missing necessary details. It is therefore important to develop a methodology that allows additional access to the literature on this topic. In this study, once the literature obtained based on a systematic search has been completed with an evidence overlay, additional intentional supplementary searches can be conducted based on the different topics of the overlay evidence.

Although some studies have argued that it is important to retrieve all the literature that needs to be included in a study at the outset (17–19), we believe that the focus on a particular topic should be progressively reasoned rather than comprehensive and systematic. Based on this consideration, we will include in the PubMed database literature related to self-management in diabetic patients retrieved during COVID-19 according to a pre-established search strategy. Then, according to the need for step-by-step reasoning, a snowball strategy will be used to gradually include more relevant literature. Previously, Yan Bo had successfully applied the snowballing strategy to investigate the public’s health needs for AI platforms during COVID-19 (20). The step-by-step reasoning process based on the use of the snowballing method to obtain literature was also used by Yan Bo in his previous studies (10, 11). This strategy makes it easier to find key literature compared to random or comprehensive searches.

In this study, Yan Bo describes the practical steps of the snowball method of acquiring supplementary literature. Supplementary literature was obtained through three steps after retrieving the seed literature.

*Step 1*: Backward snowballing, which means checking whether any of the references cited in the seed literature meet the previously established inclusion criteria, and supplementing them for inclusion if they do.
*Step 2*: Snowballing forward, this means discovering new articles citing the seed literature through Google Scholar and Scopus. Assuming that these new articles met the inclusion criteria, they were added.
*Step 3*: Theme word snowballing, in the process of reading the seed literature, if ideas and content were found that expanded on each other with the theme word of the study, keywords for the ideas and content were distilled and searched with intent in PubMed. For example, if a piece of literature mentions that a relevant point of view for self-management is that people with diabetes need to self-control their diet, then self-control diet (topic word) and COVID-19 (context word) would be used as intentional search terms, and then literature obtained based on the subjective intent after searching in PubMed would be referred to as supplemental literature obtained by the snowballing method. This use of snowball method to obtain literature based on stepwise reasoning process was also used by Yan Bo in his previous studies (10, 11).

The research criteria for the snowballing method of obtaining literature were aligned with the inclusion and exclusion criteria of the systematic review.

### 2.6 Study selection

In the process of selecting the research literature, we included the entire literature that was obtained through the search formula. Even if the topic of research in one of the literatures did not meet the qualifications of the research criteria, it did not affect our ability to distill the thinking, ideas and frameworks in the literature. We will use the distilled information to obtain more literature through snowballing strategy to fill in the gaps.

Characteristics of literature included in the systematic search process included: (1) meeting the search formula; (2) publication of the literature after 1 January 2020.

Characteristics of the literature included in the snowballing process included: (1) the literature description was based on the COVID-19 context; (2) the literature described at least “role of nurses” or “self-management.”

### 2.7 Data collection process and data extraction

Data collection and data coding were not applicable in this narrative review. We distilled the information on “role of nursing/nurses in people with diabetes” and “self-management of people with diabetes” from the seed literature only, and subsequently analyzed the literature for research methodology and study population (including study sample size). For these seed documents, we additionally obtained the number of references and the number of cited documents. Literature obtained by performing a snowballing approach based on the seed literature was summarized using a narrative synthesis.

### 2.8 Synthesis of evidence

A design-stratified synthesis of directional evidence with harvest/effect-direction plots was used, with no effect value combinations or meta-analyses. Heterogeneity was narrated as contextual versus intervention composition stratification.

There are some advantages to such a narrative synthesis. A practice framework for guiding nursing care that is broadly descriptive and scientifically sound is appropriate for nurses with an extended career scope and for people with diabetes who are intensively self-managing. A harvest/effect-direction plot and an intervention-intensity-outcome table with high levels of generalization can complement systematic reviews, meta-analyses on similar topics. This ensures reader interest, knowledge dissemination, and retains scholarly research value.

### 2.9 Risk of bias and quality assessment

This study used a narrative approach to synthesize evidence, so quality of evidence assessments and publication bias assessments were not applicable. This narrative review was evaluated using the scale for the assessment of narrative review articles (SANRA) (Table 1) (21).

**Table 1:** Scale for the assessment of narrative review articles.

Question	Points
1) Justification of the article’s importance for the readership.	2
The importance is not justified. (0 point)	
The importance is alluded to, but not explicitly justified. (1 point)	
The importance is explicitly justified. (2 points)	
2) Statement of concrete aims or formulation of questions	2
No aims or questions are formulated. (0 point)	
Aims are formulated generally but not concretely or in terms of clear questions. (1 point)	
One or more concrete aims or questions are formulated. (2 points)	
3) Description of the literature search.	1
The search strategy is not presented. (0 point)	
The literature search is described briefly. (1 point)	
The literature search is described in detail, including search terms and inclusion criteria. (2 points)	
4) Referencing	2
Key statements are not supported by references. (0 point)	
The referencing of key statements is inconsistent. (1 point)	
Key statements are supported by references. (2 points)	
5) Scientific reasoning (e.g., incorporation of appropriate evidence, such as RCTs in clinical medicine)	2
The article’s point is not based on appropriate arguments. (0 point)	
Appropriate evidence is introduced selectively. (1 point)	
Appropriate evidence is generally present. (2 points)	
6) Appropriate presentation of data (e.g., absolute vs. relative risk; effect sizes without confidence intervals)	2
Data are presented inadequately. (0 point)	
Data are often not presented in the most appropriate way. (1 point)	
Relevant outcome data are generally presented appropriately. (2 points)	
Sumscore	11

## 3 Results

### 3.1 Literature search and study characteristics

The findings of this study highlight the importance of daily self-management practices and nurses’ roles for diabetic patients during the COVID-19 pandemic.

Yan Bo ran the search formula on 1 May 2022 and found that only six literatures existed (22–27). On 10 May 2025, a total of nine studies were retrieved in PubMed based on a pre-established search strategy (22–30). Cumulative acquisition of 10 literatures on 5 August 2025 (22–31).

**Figure 1:**
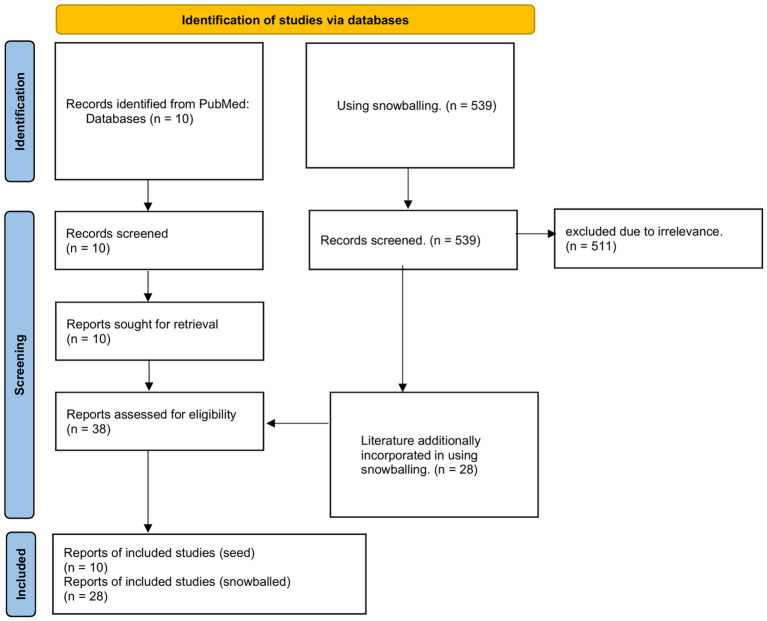
Flowchart. Identification, screening, eligibility, and inclusion stages of the review process. A total of 549 records were screened, with 38 studies meeting the final inclusion criteria.

These 10 studies constituted the seed literature for the snowballing strategy. The process of narrative inference of content based on seed literature was progressively included in the flowchart for each retrieved piece of literature that required new literature (Figure 1). We summarized the results of all seed literature in the narrative reasoning process in a table (Table 2). There were three narrative reviews, two cross-sectional observational studies, three cohort studies, one case report and one bibliometric study, totaling a cumulative total of 5,834 adult effects in these 10 pieces of literature on inductive reasoning. We then superimposed the evidence on the role of nurses and self-management using a narrative approach after categorizing them according to topic, in which a rolling ball approach is used for specific topics to supplement the retrieved literature. After applying inclusion/exclusion criteria, 38 studies [10 seed (22–31); 28 supplementary (4, 6, 32–57)] were included for analysis.

**Table 2:** Characteristics and key findings of seed literature (*n* = 10; 5,834 cases).

No.	References	Design	Population	Num ref/cited	Role of nurses	Self-management
1	Robinson Patricia (26)	Narrative literature review	NA	20/16	Control of infection prevalence; development of individualized management programs for chronic diseases; development of emergency care programs; monitoring of conditions; health promotion; establishment of home care guidelines	Home isolation; maintaining social distance
2	Chertok Ilana R. Azulay (22)	Cross-sectional observation study	810 cases with adults	28/76	Capitalizing on public concern about the COVID-19 pandemic to control diabetes by persuading smoking cessation;	Willingness to quit smoking was 6.984 times higher in diabetic smokers than in non-diabetic smokers (95% CI: 1.781–27.387, *p* = 0.005); self-perceived risk of serious infections, with each 1-point increase in the risk score being associated with an 18.5% increase in the probability of willingness to quit smoking (aOR = 1.185, 95% CI: 1.114–1.376, *p* < 0.001)
3	Moriyama et al. (25)	Cross-sectional observation study	1,749 cases with adults	9/36	Adaptation of the management model for chronic end-stage diseases during COVID-19;	Maintaining relative health reduces the incidence of serious illness; reduced willingness to treat in hospice care is associated with a lower risk of infection.
4	Chow et al. (23)	Retrospective cohort study	96 cases with adults	21/41	Use of rtCGM helps to improve care 63% (22/35); 49% of nurses report that use of PPE can healthcare resource consumption	NA
5	Lumbers Melanie (24)	Narrative literature review	NA	16/7	Stratified management of DFU infection levels can reduce capital expenditure	NA
6	Won et al. (27)	Bibliometric research	NA	30/8	Practicing the core of infection management; developing infection control strategies; preventing community infections	NA
7	Subrata and Margono. (30)	Narrative literature review	NA	86/7	Understanding patient needs to optimize care pathways	NA
8	Kim et al. (28)	Prospective cohort study	6 cases with adults	38/2	Health promotion and urging patients to carry out self-management	Self-management has a positive effect on the prognosis of diabetes. Self-management includes walking or moderate-intensity exercise, weight management, blood pressure management, blood glucose control, dietary control, control of calorie intake, regular measurement of heart rate, assessment of wakefulness, and maintaining a non-smoking habit.
9	Smith et al. (29)	Retrospective cohort study	3,172 cases with adults	45/9	COVID-19 Initiation of telemedicine during an outbreak: messaging, telephone and video consultations	Use of digital teleconsultation and regular consultations
10	Liston et al. (31)	Case report	1 case with adult	32/0	Routine care	A probable relationship between diabetic ketoacidosis and coronavirus disease 2019, resulting in extreme hyperglycemia.

**Figure 2:**
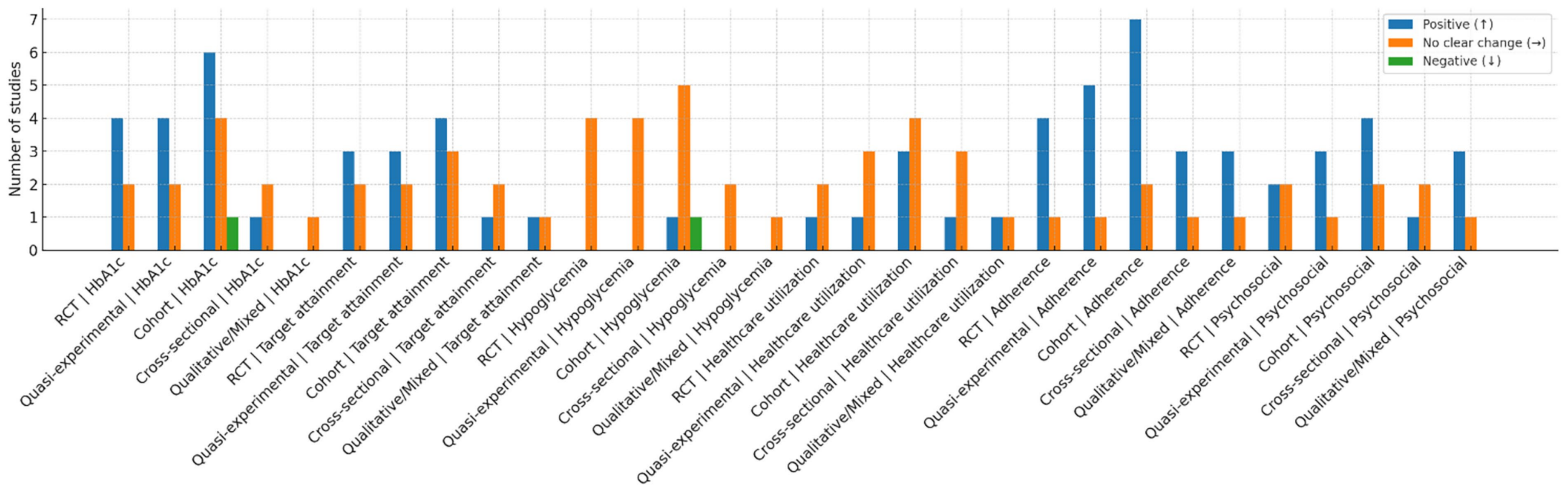
Harvest/effect-direction plot by study design and outcome domain. Bars show the number of included studies in each combination of study design and outcome domain that reported a favorable (↑), no clear change (→), or unfavorable (↓) direction of effect. Study designs were coded as randomized controlled trials (RCTs), quasi-experimental studies, cohort studies, cross-sectional studies, and qualitative/mixed-methods studies. Outcome domains were HbA1c, treatment-target attainment, hypoglycemia, healthcare utilization, adherence, and psychosocial outcomes (distress, self-efficacy). Directional coding followed the pre-specified narrative-synthesis approach; no effect sizes were pooled. Each of the 38 included studies could contribute to more than one domain but was coded once per relevant domain using a predefined hierarchy; disagreements were resolved by adjudication. As shown, favorable directions are most frequent for HbA1c and adherence across experimental and observational designs, while hypoglycemia is predominantly neutral and utilization outcomes are mixed. The figure is descriptive and should not be interpreted as a quantitative estimate of effect. This is drawn by Figdraw.

This narrative mini-review is intended for nurses and volunteer individuals with diabetes deployed to emergency response frontlines. These groups require broad, evidence-based knowledge to inform their practice. Here, we summarize consistent findings, key gaps, and reflections on the included literature. We conducted a cumulative, critical synthesis of 38 pieces of evidence. Three studies reported that nurse-led telemonitoring during COVID-19 was associated with reductions in HbA1c of up to 0.86% among people with diabetes (41, 49, 54). Four studies demonstrated consistent outcomes regarding nurses’ provision of psychosocial support, with the magnitude of benefit varying by age and duration of contact (38, 47, 50, 57). Four studies highlighted key gaps stemming from the underrepresentation of vulnerable populations, including individuals of low socioeconomic status, ethnic minorities, older adults, and those with multiple comorbidities (29, 36, 45, 54). Our assessment of study designs identified 2 randomized controlled trials (49, 50), 6 reviews (4, 6, 37–39, 54), and 30 observational studies. Cumulatively, 24 studies reported outcomes directly tied to the COVID-19 context (4, 6, 22–27, 29–37, 41, 43, 45–47, 54, 57). This confirms the success of our two-stage literature retrieval approach, which combined systematic searches and snowballing. Additionally, literature published outside the period of high COVID-19 prevalence remains relevant to practice during the pandemic. The use of 38 pieces of evidence to develop a framework for expanded care applicable to COVID-19 and future public health crises aligns with the foundational aims of our research (Figure 2).

Data were obtained from the corresponding literature (22–31). For the literature review type of studies, the role of nurses and self-management, they were summarized using the topic distillation method. For the original research type of literature, it was summarized using correlates, risk factors or insights from the research. Real-Time Continuous Glucose Monitoring (rtCGM); personal protective equipment (PPE); diabetic foot ulcer (DFU); NA indicates not available.

### 3.2 Self-management of diabetes

The content of digital care services provided by nurses to diabetic patients during the COVID-19 pandemic remains consistent with that provided during non-COVID-19 periods (6). The American Diabetes Association recommends that healthcare providers deliver diabetes care through a multi-module approach encompassing medical assessment, lifestyle management, blood glucose control, medication therapy, and prevention of diabetes complications. This article will primarily adopt a narrative descriptive approach to explore digital care for diabetes during the COVID-19 pandemic, organised according to these task modules.

#### 3.2.1 Control and detection of blood sugar

During the COVID-19 pandemic, diabetic patients must maintain regular blood glucose monitoring habits to ensure that blood glucose levels remain within a stable range. From the patient’s perspective, in regions under COVID-19 lockdown or during medical isolation, patients’ previous regular blood glucose monitoring habits may be disrupted. This may be due to difficulties in purchasing or replenishing blood glucose monitoring devices and needle-related consumables (32). This can indirectly lead to uncontrolled blood sugar levels in diabetes patients. Considering this, nurses who have access to diabetes patients may take certain measures to address the issue. First, nurses will contact diabetes patients via phone or WeChat and strongly recommend purchasing blood sugar monitoring materials and diabetes medications online. Second, nurses will proactively assist patients in adjusting their daily blood sugar monitoring frequency based on factors such as age, current comorbidities, and other risk factors affecting blood sugar control (33). Additionally, nurses require diabetic patients undergoing COVID-19-related closed-loop blood glucose control to collect fingerstick blood samples daily for monitoring. Evidence supports this approach. Patients with diabetes who contracted COVID-19 and completed planned treatment may still have suboptimal health outcomes even after successful discharge (33). This highlights the significant importance of actively monitoring blood glucose levels within a stable range for diabetic patients with COVID-19 infection.

The blood glucose control pathway established by the aforementioned nurses primarily benefits diabetic patients in two types of situations.

In the first scenario, the patient’s blood glucose levels are relatively stable. Here, relatively stable blood glucose levels are defined as fasting blood glucose levels below 6.9 mmol/L or glycated haemoglobin HbA1c levels below or equal to 6.5%. This may be because the patient has received good education and is therefore able to take oral hypoglycaemic drugs regularly even without supervision during the COVID-19 pandemic. Diligent nurses adjust the monitoring frequency for this group of patients with stable blood glucose levels to once or twice a week, with monitoring conducted both fasting and postprandial (34).

In the second scenario, patients have poor blood glucose control. Patients of this type may experience intermittent hypoglycaemia because they are insensitive to the effects of insulin injection therapy. However, according to information provided by nurses in interviews, patients with poor blood glucose control generally lack self-management awareness. Interestingly, the effects of poor self-management awareness are amplified during the COVID-19 pandemic due to lockdowns. Dedicated nurses provide self-management education to such patients and adjust their blood glucose monitoring frequency to four times daily for one week until stabilisation; monitoring times are adjusted to fasting, before lunch, before dinner, and before bedtime. These patients are eligible for free telemedicine consultations. If their blood sugar becomes uncontrolled or they face other challenges, they can notify their responsible nurse to initiate a telemedicine consultation (35). This allows diabetic patients lacking self-management awareness to promptly receive feasible guidance strategies from healthcare providers.

#### 3.2.2 Diet control

There is no fundamental difference between dietary control for diabetic patients during the COVID-19 pandemic and during non-pandemic periods (36). Nurses will categorise dietary control plans based on the body mass index (BMI) of diabetic patients. For diabetic patients with a BMI assessment result of obesity, the ideal calorie intake is 20 kcal/kg. For diabetic patients with a BMI assessment result of non-obesity, the maximum ideal calorie intake is 25 kcal/kg. For diabetic patients with kidney disease, nurses recommend that dietary fibre account for 45% of total intake, and that total fat intake does not exceed 30% per day. To accommodate diabetic patients who lack self-management capabilities, nurses will kindly prepare an electronic version of the plate method and send it to patients via the internet, allowing patients to follow the plate method to perform daily dietary control tasks. The plate method refers to eating only one plate of food per meal, with half of the plate containing vegetables, one-quarter containing protein-rich foods, and the remaining quarter containing carbohydrate-rich foods. The greatest challenge in dietary management for diabetic patients during the COVID-19 pandemic was the inability to obtain fresh green vegetables. Nurses proactively assist patients in contacting online vegetable vendors for delivery.

#### 3.2.3 Activity exercise

During the COVID-19 pandemic, diabetic patients were unable to go to parks for physical exercise due to lockdowns and home quarantine. Regular physical exercise is a key factor in maintaining the health of diabetic patients (37). Nurses develop home exercise programmes to address this challenge and require patients to follow them. For patients with fitness equipment at home, nurses tailor plans based on the available equipment. For example, using a treadmill for 60 minutes daily, with exercise intensity limited to a heart rate above 100 without feeling overly fatigued. If patients lack both fitness equipment and the physical fitness required for moderate-intensity bodyweight exercises, nurses recommend light aerobic exercises. For instance, patients can spend 15 minutes daily walking up and down stairs between the first and top floors, or perform household chores. These light aerobic exercises help maintain the daily basic exercise requirements for diabetic patients (37, 38). It is particularly important to note that diabetic patients with a history of heart disease should wear a smartwatch that monitors heart rate and blood pressure in real time, and should have a family member accompany them during exercise to prevent delayed detection of hypoglycaemia or sudden heart attacks (39).

#### 3.2.4 Medication precautions

Guidance on medication use for diabetes patients during the COVID-19 pandemic has been relatively straightforward. Every newly diagnosed diabetes patient receives medication education at the hospital outpatient clinic. After discharge, diabetic patients are regularly followed up by a nurse via telephone to monitor medication adherence and medication stock levels. If patients have any questions about their medications while at home, they can notify the nurse to initiate a remote consultation and receive professional guidance from an endocrinologist (35). If a patient’s medication supply is insufficient and they are unable to replenish it due to lockdown restrictions, the responsible nurse will confirm the required medications online, obtain approval for the prescription from an endocrinologist, and then coordinate with a dedicated team to deliver the medications to the patient’s home.Of course, during the lockdown, the most frequent reminders from nurses were about preventing COVID-19 infection. As of 2022, there is no substantial evidence to suggest that anti-diabetic medications are equally effective in treating COVID-19-infected diabetic patients compared to diabetic patients without COVID-19. Although angiotensin-converting enzyme (ACE) inhibitors and angiotensin receptor blockers (ARBs) are theoretically beneficial for controlling diabetes in patients with COVID-19 infection, this is not supported by experimental data. Additionally, to prevent accidental hypoglycaemic coma, nurses actively coordinate with endocrinologists to assist patients in adjusting their hypoglycaemic medications and insulin doses. If diabetic patients experience any adverse reactions while taking hypoglycaemic medications or administering insulin, they are immediately informed to the responsible nurse.For diabetic patients infected with COVID-19, nurses will promptly contact the emergency hotline for assistance, followed by comprehensive treatment. Of course, the rates of glucocorticoid therapy among diabetic patients with COVID-19 in the ICU and non-ICU settings are 72.2% and 44.9%, respectively. Previous experience suggests that such glucocorticoid pulse or adjunctive therapy often induces more severe clinical symptoms. Although chloroquine-based drugs also have hypoglycaemic and immunomodulatory effects, animal studies have shown that they can cause an increase in serum insulin levels (40). These experiences in treating COVID-19 infections suggest that outcomes for diabetic patients after infection are often unfavourable. Therefore, nurses will particularly remind patients to prevent COVID-19 infection.

#### 3.2.5 Diabetes-related education

Due to lockdowns during the COVID-19 pandemic, it was not feasible for diabetic patients to visit hospitals regularly for follow-up treatment (41). To address this challenge, nurses initiated telemedicine services via telephone or WeChat. Responsible nurses obtained a list of long-term diabetic patients requiring follow-up from endocrinologists and then conducted online education sessions once a month. Prior to each online education session, responsible nurses invited each diabetic patient on the follow-up list to participate in the online meeting via telephone.A review synthesising evidence from multiple studies demonstrated the effectiveness of telemedicine (41). During non-COVID-19 periods, telemedicine treatment significantly reduced HbA1c levels by 0.37% in diabetic patients. This effect was consistent in clinical observations of remote guidance (HbA1c reduction of 0.31%). This telemedicine approach shows no selectivity in treatment outcomes for both type 1 diabetes (HbA1c reduction of 0.12% to 0.86%) and type 2 diabetes (HbA1c reduction of 0.01% to 1.13%).To protect patient privacy, nurses recommend that patients actively inquire about the disposition of patient privacy records generated during their first remote education session conducted primarily via video calls. These records may be stored in an anonymised format or retained under the patient’s real name. If the patient is under 18 years of age, consent from their parents must be obtained. This allows for real-time communication regarding issues related to the patient’s daily self-management during the video call-based remote education session. For example, if a diabetes patient develops a wound on their foot and suspects diabetic foot, they can use video or photos to inform the endocrinologist, who can then provide targeted guidance (37).

#### 3.2.6 Prevention of COVID-19

As mentioned in our discussion above, people with diabetes are at higher risk of contracting COVID-19 and developing severe cases. Until the local disease control centre announces that COVID-19 has been eradicated, nurses recommend that people with diabetes continue to practise good self-protection, avoid contact with COVID-19 transmission routes, and maintain a safe distance from other people.Nurses will regularly call diabetic patients to develop personalised diabetes management plans (42), which will help patients prevent COVID-19 infection. If patients suspect that they have contracted COVID-19, they should immediately report it to the nurse in charge and seek treatment (33). If patients have unavoidable circumstances that require them to go to crowded places, they should wear masks that block aerosols at all times.

#### 3.2.7 Population-specific, situation-specific diabetes care

During the COVID-19 pandemic, diabetes management has inevitably changed. It should be personalised based on the patient’s age, comorbidities, and socioeconomic status. For example, older adults or those with chronic kidney disease may require different treatment approaches than younger, healthier individuals (43). Therefore, specific situations require specific responses. The following sections will discuss diabetes management in specific populations or situations.

For children or adolescents newly diagnosed with type 1 diabetes, a face-to-face consultation model is recommended (44). Patients with type 1 diabetes and their families should visit a diabetes clinic to initiate insulin therapy. Healthcare providers should ensure that patients and their families receive diabetes education, with a focus on insulin therapy, signs/symptoms, and management of hypoglycaemia and ketoacidosis (35). For follow-up of patients with type 1 diabetes, ketoacidosis testing should be recommended when hyperglycaemia occurs (45).

Patients with gestational diabetes mellitus (GDM) should receive face-to-face insulin counselling at their first visit (46). Patients should receive targeted diabetes education and current condition management to enable proper lifestyle management (35, 47). GDM patients may require fine-tuning of insulin doses and should be followed up using telemedicine (41).

Additionally, older diabetic adults are more prone to worsening glycaemic control due to elevated blood glucose levels (32, 45). During lockdown and isolation periods, limited access to medical care may lead to hyperglycaemia or hypoglycaemia, exacerbating glycaemic instability (32). This situation can further worsen the condition of older diabetic adults and lead to complications such as diabetic ketoacidosis, infections, hyperosmolar non-ketotic diabetic coma, and cardiovascular diseases, particularly among older diabetic adults living alone (45). Therefore, it is recommended that such patients maintain regular contact with healthcare professionals and seek assistance promptly when needed.

When diabetic patients experience symptoms such as excessive sleepiness, vomiting, chest pain, shortness of breath, or weakness in the limbs, these should be considered emergency situations, and medical personnel should be contacted immediately for assistance. Additionally, diabetic patients with complications such as foot ulcers, gangrene, severe hypoglycaemia, gastroenteritis, or other COVID-19-related infections require specialised treatment. In such cases, patients should promptly seek medical attention at a hospital or clinic or be admitted for treatment. Medical personnel should continuously monitor the patient’s vital signs/symptoms and take initial measures to schedule an appointment at a hospital/clinic (41). For confirmed COVID-19 diabetic patients receiving intensive care, blood glucose monitoring should be intensified, and adverse drug reactions should be identified early. It has been reported that COVID-19 patients with diabetes are twice as likely to be admitted to the ICU and receive intensive care compared to uninfected patients (4).

### 3.3 The role of nurses on diabetic patients

#### 3.3.1 Nurses as science popularizers

Patient education is the foundation of diabetes management. Educating patients about diabetes, the importance of diet and exercise, medication adherence, and self-monitoring of blood glucose can enable them to effectively control their condition.

Nurses play a key role in educating diabetes patients on self-management within the healthcare system. The reason for this task allocation is that when nurses are involved in diabetes health education, patients develop a positive interest in learning about diabetes (42). This indirectly increases the positive impact on recovery. This effect is more pronounced in patients who are eager to acquire more professional knowledge to stabilise their blood sugar levels (48). Bostrom’s study on the role of diabetes specialist nurses demonstrated the importance of nurses’ roles in educating diabetes patients (42). During the COVID-19 pandemic, nurses seemed to play a more teacher-like role in the implementation of digital nursing. In China, a civilised nation with a cultural heritage spanning 5,000 years, people generally respect their older adults and are willing to accept the knowledge they impart. Diabetes patients understand that the purpose of this knowledge transfer is to help themselves, not others. Deborah J Wexler and colleagues’ research explored the risk factors for diabetes, with elevated C-reactive protein levels increasing the risk of diabetes onset (49). This evidence indirectly highlights the importance of nurses in the role of educators. During the COVID-19 pandemic, diabetes patients often struggled with self-management due to a lack of professional knowledge, which can exacerbate endogenous inflammation within the body, typically manifesting as anxiety. Based on our team’s clinical and nursing experience, we observe that patients often exhibit elevated C-reactive protein levels after blood sample testing. A study by Raballo et al. indicated that diabetic patients receiving group care had greater benefits in terms of diabetes metabolism (50). This may be because diabetic patients need more support from patient teams. Notably, high blood sugar levels in diabetic patients were associated with negative attitudes in conversations in the results of Raballo et al.’s study.

These findings suggest that diabetes patients themselves need social support for self-management of their disease, rather than simply drug treatment. Nurses, in their role as teachers, may be able to fill this need.

#### 3.3.2 Nurses as professional caregivers

Does the extended role played by nurses in nursing practice during the COVID-19 pandemic align with the regulatory framework for registered nurses? Nursing and Midwifery Council (58) and International Council of Nurses (59) defines Advanced Practice Nurses (APNs) as skilled nurses who can perform various roles in the care of diabetic patients. Clearly, nurses playing an extended role in nursing practice aligns with regulatory requirements. In the context of COVID-19, the type of assistance sought by diabetes patients is more akin to a learning disability. Blakemore (51) reported on her past experience seeking pain care from a nurse, which greatly inspired her career goals (51). We firmly believe this is the best evidence of nurses assisting diabetes patients during the COVID-19 pandemic from a learning disability perspective.

The view in the literature is that nurses are referred to as healthcare professionals not only because they can play the role of a teacher or mother in caring for patients, but because they can participate in managing the care plans for specific medications for diabetic patients (52). Carey et al. found that over two-thirds of specialist nurses in the UK prescribe medications for common diabetes complications, even if this requires an additional 20% of their time. These nurses are willing to dedicate themselves to this task, driven by their noble belief in saving lives and alleviating suffering (53). Such cases also exist in China. According to Dr. Zhixiong Zhuang, senior nurses in large hospitals in China’s Huaxi region also have prescribing authority for medications. A 2020 meta-analysis indicated that HbA levels in diabetes patients have the potential to be effectively reduced through telemedicine (54). This suggests that during the COVID-19 pandemic, if nurses possess more knowledge about prescribing diabetes medications, it could facilitate improvements in HbA levels through telemedicine.Another perspective in the literature emphasises the importance of nurses screening for diabetes and diabetes-related complications. This reflects the core professional responsibilities of Advanced Practice Nurses (APNs). For example, after assessing a patient’s ocular and foot complications, nurses are required to write a report and submit it to an endocrinologist (43). This process may alleviate the endocrinologist’s challenges in collecting clinical data. In this scenario, nurses function more as clinical assistants (55, 56). In other words, the nursing tasks performed by nurses are essentially part of the clinician’s medical orders. Some also view nurses as intermediaries between clinicians and diabetes patients. This is because clinicians often base their initial clinical decisions on nurses’ preliminary assessments of patients (55).Overall, nurses purposefully plan and collaborate with clinicians and diabetes patient groups to deliver diabetes care.

#### 3.3.3 Nurses’ psychological counseling role for diabetic patients

The literature suggests that nurses provided psychological counselling services to diabetic patients during the COVID-19 pandemic, which helped alleviate patients’ negative emotions. Rita Forde’s 2021 study conducted by the European Diabetes Nurse Foundation Survey Alliance provides evidence for this. Among the nurses surveyed, 82% reported that diabetic patients sought nursing assistance due to anxiety (57). Dr. Zhixiong Zhuang affirmed this view based on his medical practice experience. During the COVID-19 pandemic, every hospital in China established mental health helplines for patients to use. This indicates that digital nursing during the COVID-19 pandemic placed greater emphasis on social relationships.

#### 3.3.4 Nursing roles and intervention taxonomy

Across studies, nurse involvement coalesced into four modules: (1) structured education: dietary counseling, insulin and oral agent literacy, sick-day protocols, device onboarding; (2) remote monitoring and feedback: review of uploaded capillary or CGM data, algorithm-guided flags, medication titration in collaboration with physicians; (3) adherence support: scheduled tele-visits, SMS reminders, recovery of missed uploads, barrier troubleshooting; and (4) psychosocial care: brief screening for distress, motivational interviewing, caregiver engagement, and mental-health referral.

Programs operationalizing more than 2 modules at a cadence of at least monthly demonstrated more consistent favorable directionality in HbA1c.

We attempt to summarize specific examples of nurses’ role in diabetes patients’ self-management in the form of a table (Table 3). These specific intervention programs are replicable extensions of the four intervention models mentioned above.

**Table 3:** Mapping nurse-led digital diabetes care modules, intervention intensity, and outcome directions during the COVID-19 era (directional evidence only).

Intervention module (replicable description)	Intensity	Outcome directional trends	Supporting studies	Brief evidence points
Remote follow-up/remote monitoring (including CGM/FGM, remote injection guidance, and process reengineering)	Moderate	↑	(32, 35, 47, 54)	During the lockdown period, adult T1D using FGM/CGM showed TIR↑ and estimated HbA1c↓; video/telephone guidance on insulin injection and follow-up were feasible, with satisfaction and adherence↑; obstetric/glucose metabolism follow-up quality control programs improved detection and follow-up rates; reviews showed that most digital interventions led to slight improvement in HbA1c.
Remote follow-up/remote monitoring (acceptability/utilization disparities)	Small	→	(23, 31, 41)	Healthcare providers have a high perception and acceptance of rtCGM real-time data, but implementation details vary; the pandemic has promoted remote care, yet it faces barriers in primary-level deployment; there exist population and structural disparities in the use of remote consultation (with unequal accessibility).
Education and self-management support (group education/group clinic)	Moderate	↑	(50, 55)	Group education/group clinics are superior to routine follow-up (metabolic control, knowledge and self-management behaviors, satisfaction↑); frontline nurses’ participation in diagnosis/screening processes can improve the timeliness of diagnosis and pathway efficiency.
Education and self-management support (pump/technology brief counseling, attitudes and competencies)	Small	→	(44, 52, 56)	“15-min pump consultation’ and other technical education facilitate processes and skills, but short-term clinical outcomes are not clear; primary-level nurses” attitudes/concerns, knowledge and role definition affect the achievement of education.
Nurse-led medication delivery/process	Moderate	↑	(53)	Nurse-led medication delivery services are acceptable in terms of safety and accessibility, reducing outpatient contact and process bottlenecks.
Foot/community care (infections and ulcers)	Small	→	(24, 30)	Community nurses’ roles in osteomyelitis/diabetic foot identification, triage and chronic disease follow-up are emphasized; during the pandemic, a holistic care framework integrated with needs theory was proposed, but lacks quantitative outcomes.
Inpatient/transitional care (critical illness/end-of-life related)	Moderate	→	(25)	Multidisciplinary teams’ communication, symptom management, and family support during the transition period of critically ill inpatients are regarded as critical; early data focus on feasibility and process descriptions.
Pregnancy/perinatal period (including GDM self-management and follow-up)	Moderate	↑	(46, 47)	During the pandemic, obstetric quality improvement projects and process reshaping improved GDM and postpartum glucose tolerance follow-up rates; key points of GDM self-management in the context of home isolation were advocated.
Lifestyle intervention (dietary quality/physical and psychological intervention)	Moderate	↑	(38)	Higher dietary quality is associated with lower risk of COVID-19 infection/severe disease; mind–body interventions such as yoga may yield benefits in metabolic and psychological indicators.
COVID-19 with diabetes outcome risks (large population datasets)	High	↓	(33, 45)	COVID-19 and new-onset T1D risk↑; pre-existing T1D patients: DKA risk↑; T2D/hyperglycemia and severe COVID-19 association degree↑.
COVID-19 with diabetes outcome risks (small sample/case series)	small	→	(34)	A small number of cases suggest blood glucose fluctuations during infection, but directions and magnitudes vary, with unstable evidence.
Medication safety (diabetes with COVID-19)	Moderate	↓	(40)	Comprehensive assessment suggests that risks of complications/drug interactions with some medications need to be weighed, emphasizing individualization and monitoring.
Mechanisms and contextual evidence (inflammation/comorbidity reviews)	Small	→	(4, 6, 49)	The link between inflammation and insulin resistance/metabolic abnormalities supports the framework of metabolic vulnerability in the context of the pandemic; comprehensive reviews and annual reviews summarize the association between comorbidities and outcomes, providing a theoretical basis for clinical risk stratification.
Guidelines/consensus/rehabilitation pathways (endocrinology/cardiopulmonary rehabilitation/nursing joint statements)	Small	→	(37, 39, 57)	Joint statements on key points of endocrine management, exercise/rehabilitation frameworks, and patient-reported outcome measures (PROMs) in the context of the pandemic can provide reference for pathways and quality evaluation, but are not evidence of interventional outcomes.
Nursing workforce and professional roles (role diversity/psychological burden/institutional environment)	Small	→	(42, 43, 48, 51)	Nurses’ contributions and stress during the pandemic were systematically stated
Community health behaviors (smoking risk perception)	Small	→	(22)	Perception of infection risk influences health behaviors such as smoking, indicating entry points for community health education.
Nursing informatics/topic mining (field context)	Small	→	(27)	Text mining-based nursing research hotspots (including diabetes-related topics) provide clues for developing training and research agendas, not direct clinical outcomes.
Emergency nursing processes/case review (ketoacidosis)	Small	→	(31)	Case reviews of DKA emergency care are used for process reflection and safety improvement, not non-controlled outcome research.
Primary health/community chronic disease management and SARS-CoV-2	Small	→	(26)	Continuity and risk balance of community chronic disease management under the pressure of infectious diseases have been discussed, providing context for primary-level integrated care.
Cross-disease DSME/S (self-management implications for stroke survivors)	Moderate	↑	(28)	Structured DSME/S have good feasibility/satisfaction in the chronic phase of stroke, and have translatable process value for the framework of diabetes self-management.

Arrows denote directional trends relative to baseline or usual care: ↑ improvement; → no consistent change; ↓ worse outcome; magnitude labels (small/moderate) reflect narrative judgment from study designs and reported metrics, not pooled effects. Intensity combines touchpoint frequency, data-return completeness, and feedback latency (lower is faster). CGM, continuous glucose monitoring; FGM, flash glucose monitoring; GDM, gestational diabetes mellitus; DSME/S, diabetes self-management education and support; T1D, type 1 diabetes; T2D, type 2 diabetes; DKA, diabetic ketoacidosis; PROMs, patient-reported outcome measures.

## 4 Discussion

We explored the digital nursing model implemented by nurses and self-management by diabetes patients in the context of COVID-19. The question that both finally addressed during COVID-19 was how to stabilise and control blood sugar levels.

For diabetes patients themselves, the key to self-management is to acquire more diabetes-related educational knowledge and then complete the tasks of the care module driven by self-management awareness. Diabetes patients must regularly monitor their blood sugar levels and take oral hypoglycaemic drugs regularly. During the COVID-19 lockdown, diabetes patients should consciously and proactively complete some basic exercises at home that they are capable of doing to stabilise their diabetes-related basal metabolic rate.

However, during the COVID-19 pandemic, a group of diabetes patients lacking self-management skills indirectly developed a nurse-led digital care model. Nurses need to actively provide resources and professional guidance to these groups via telephone or the WeChat app. The primary objective of this digital care model is to cultivate healthy behavioural habits among diabetes patients, thereby enabling sustainable self-management of blood glucose levels. Clinical practice experience has demonstrated that nurses can indeed bridge this gap through virtual or telemedicine consultations. This approach not only effectively enhances the continuity of diabetes care but also reduces the risk of patients contracting COVID-19 due to exposure in high-risk areas.

This digital care module developed by diabetes patients during the COVID-19 pandemic offers new insights into the professional development of nurses. In previous care models, most nurses focused primarily on face-to-face communication and nursing education for hospitalised patients. Interestingly, nurses can now establish specialised telemedicine teams to provide additional support and resources to diabetes patients who require it, thereby addressing the shortcomings of traditional care models.

### 4.1 Pandemic context and heterogeneity

The background of this topic study is the COVID-19 pandemic. During the early stages of the COVID-19 outbreak (2020), the primary tool nurses used for digital nursing was the telephone. During this phase, healthcare workers, due to a lack of understanding of the characteristics of COVID-19 virus infection, tended to provide remote care models with lower technical complexity. As understanding of the characteristics of COVID-19 infection gradually improved, COVID-19 prevention efforts entered a stable phase (2022–2024). During this stable phase, nurses could provide hybrid expanded digital care models, such as in-person or video calls. The heterogeneity in this research stems from the differing lockdown policies across regions. These valuable experiences were gained through continuous exploration by healthcare providers and the public during the COVID-19 pandemic.

### 4.2 Implications of the study

Help develop strategies for self-management of diabetes; confirm the role of nurses in the above process. Evidence from the pandemic indicates that frontline nurses will face strategic shifts in their roles moving forward. Nurses will need to integrate curricular education with telemedicine and develop proficiency in virtual assessment technologies. This, in turn, means that individuals with diabetes will gain greater access to resources from publicly funded free healthcare, enabling them to enhance their self-management capabilities.

### 4.3 The added value of research

During the COVID-19 pandemic, combining comprehensive daily self-management programmes with attentive support from nurses is crucial to maintaining and improving the health of patients with diabetes. As the healthcare sector continues to evolve, these insights will help guide best practices and develop interventions to protect the health of patients with chronic diseases in future public health threats.

## 5 Conclusion

During the COVID-19 pandemic, the unwavering dedication of nurses accelerated the expansion of their professional scope. This expansion presents both an opportunity for nurses to diversify their roles within the healthcare sector and a means to enhance the self-management capabilities and overall well-being of individuals with diabetes. These broadened professional boundaries can serve as a valuable reference for frontline response efforts in potential future infectious disease pandemics.
